# Chemically Induced Extracellular Ice Nucleation Reduces Intracellular Ice Formation Enabling 2D and 3D Cellular Cryopreservation

**DOI:** 10.1021/jacsau.3c00056

**Published:** 2023-04-25

**Authors:** Kathryn A. Murray, Yanan Gao, Christopher A. Griffiths, Nina L. H. Kinney, Qiongyu Guo, Matthew I. Gibson, Thomas F. Whale

**Affiliations:** † Department of Chemistry, University of Warwick, Gibbet Hill Road, Coventry CV4 7AL, United Kingdom; ‡ Division of Biomedical Sciences, Warwick Medical School, University of Warwick, Gibbet Hill Road, Coventry CV4 7AL, United Kingdom; § Department of Biomedical Engineering, Southern University of Science and Technology, Shenzhen, Guangdong 518055, China; ∥ Department of Aquatic Resources, Institute of Marine Research, Swedish University of Agricultural Sciences, Turistgatan 5, 453 30 Lysekil, Sweden

**Keywords:** Cryopreservation, 3D cell assemblies, ice nucleation, polysaccharides, chemical control of ice formation

## Abstract

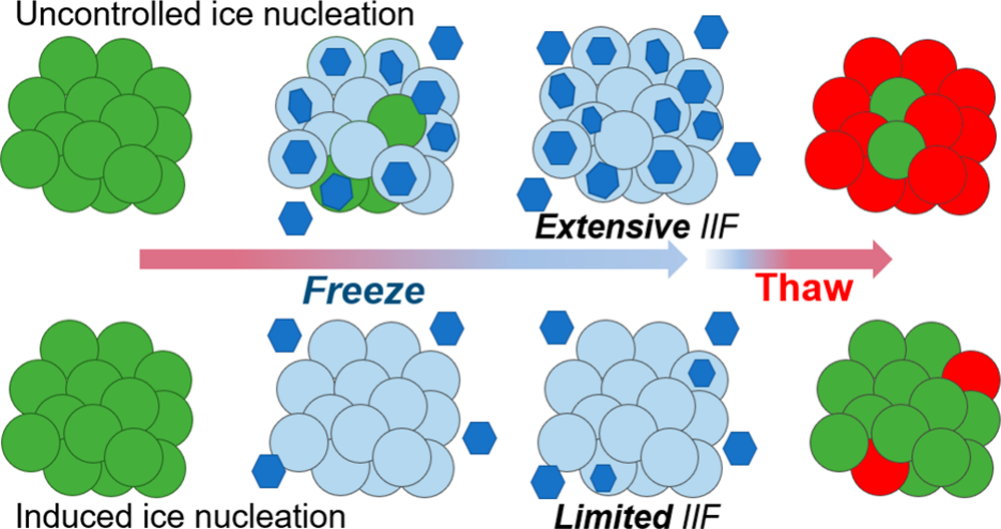

3D cell assemblies such as spheroids reproduce the in vivo state more accurately than traditional 2D cell monolayers and are emerging as tools to reduce or replace animal testing. Current cryopreservation methods are not optimized for complex cell models, hence they are not easily banked and not as widely used as 2D models. Here we use soluble ice nucleating polysaccharides to nucleate extracellular ice and dramatically improve spheroid cryopreservation outcomes. This protects the cells beyond using DMSO alone, and with the major advantage that the nucleators function extracellularly and hence do not need to permeate the 3D cell models. Critical comparison of suspension, 2D and 3D cryopreservation outcomes demonstrated that warm-temperature ice nucleation reduces the formation of (fatal) intracellular ice, and in the case of 2/3D models this reduces propagation of ice between adjacent cells. This demonstrates that extracellular chemical nucleators could revolutionize the banking and deployment of advanced cell models.

Cryopreservation is essential for all biomedical and fundamental cell biology and is the key tool to ensure delivery of cell-based therapies and other biologics.^1−4^ The main aim in cryopreservation is to slow cellular processes to enable long-term storage while retaining cell function post-thaw, but this requires the addition of cryoprotectants to mitigate the damaging effects of ice formation/growth on cells. For mammalian cells, the most common additive is DMSO, which helps dehydrate the cells and protects them from osmotic damage. Recently, innovative cryoprotectants that mitigate damage pathways not addressed by DMSO have emerged,^5^ such as ice recrystallization inhibitors,^6−8^ macromolecular cryoprotectants^9,10^ and apoptosis inhibitors.^11,12^ While numerous compounds have now been examined for cryoprotective effects many questions regarding the role of chemical additives in cryobiology remain open.^5^

A long-standing challenge in cryobiology is the control of ice nucleation. Aqueous solutions tend to supercool below their equilibrium melting point, especially in smaller volumes. In microlitre droplets, water does not tend to nucleate until below approximately −20 °C^13^ and in multiwell plates (used for handling cells) until −15 °C.^14,15^

Extracellular ice formation at warm temperatures enables cellular dehydration during cryopreservation by allowing mass transfer from the thermodynamically metastable, supercooled cell interior to the thermodynamically stable extracellularly formed ice.^16^ This removal of water from cells reduces the likelihood of fatal intracellular ice formation (IIF).^17^ The original studies on controlled rate cryopreservation employed seeding with ice crystals to ensure ice formed extracellularly.^17^ This method is difficult to implement at scale, and is often unnecessary for cryopreservation of milliliter scale volumes, in which ice tends to form at warm temperatures even in the absence of deliberately introduced ice nucelatiors.^14,18^ The optimum temperature for ice nucleation is not well established and may vary by cell type. For instance, Lauterboeck et al. found that a nucleation temperature of −10 °C was optimal for mesenchymal stromal cells,^19^ while it has been shown very recently that nucleation at the melting point was most beneficial for cryopreservation of human hepatocyte carcinoma cells.^18^ Nevertheless, it is clear that deep supercooling during cryopreservation impairs cells recovery.

Cryopreservation of cells in smaller volumes of liquids often proves challenging, however. Daily et al. demonstrated that induced ice nucleation in the extracellular space can increase post-thaw recovery of adherent primary cell cultures in 96-well plates from 30 to 58%.^14^ This is remarkable, as it shows that stimulated ice nucleation in the extracellular environment leads to protection of the intracellular environment. Such a design principle is appealing as questions around equilibration time, cellular uptake and toxicity can be easily mitigated, in contrast to intracellular delivery of cryoprotectants.^20−22^ There are, however, few accessible materials which can nucleate ice at warm temperatures. Ice nucleating proteins from *Pseudomonus syringae* are potent nucleators of potential cryobiological utlity,^23^ but have not been isolated pure, due to the significant (insoluble) transmembrane domains.^24,25^ Feldspar can nucleate ice at warm temperatures^26^ as can silver iodide,^27^ but these are not readily soluble in aqueous solutions and require segregation from the cells.^28^ Hence, these materials are not easy to deploy in cryopreservation. Physical stimuli, such as electrofreezing^29^ can induce ice nucleation but are not practical to deploy and potentially impose additional stress to the cells undergoing cryopreservation. A unique example of a soluble ice nucleator is a polysaccharide (which has not yet been fully characterized) present on the surface of some pollen grains.^30−33^ Murray et al. demonstrated that pollen washing water (PWW) is easily sterilizable (using filtration) and, as it is soluble, could be supplemented to DMSO-based cryopreservation, leading to significant increases in post-thaw recovery of adherent cell monolayers at a range of freezing rates.^15^

Cellular spheroids (and organoids) more accurately reproduce the in vivo niche than (2D) cell monolayers.^34,35^ For example, hepatocyte spheroids predict in vivo toxicological responses more accurately than monolayers and hence can play a role in reducing animal testing.^36,37^ The FDA modernization act 2.0 has removed the requirement for animal testing in drug discovery, where suitable cell models are available.^38^ During cryopreservation of 3D cell models, uncontrolled ice nucleation leads to widespread damage due to extensive cell–cell contacts, which enable fatal intracellular ice to propagate.^39^ Due to this, DMSO cryopreservation of spheroids does not always give high recovery/viability,^40,41^ and hence complicates standardization and replication.^42^ Induced ice nucleation has been shown to increase recovery to >80% (with high viability) but protein secretion was reduced compared to fresh.^43^ There is a clear need to improve methods for 3D cell storage and distribution.

Here we demonstrate that chemically triggered extracellular ice nucleation reduces intracellular ice formation when supplemented into DMSO cryopreservation media. For monolayers and spheroids, which have extensive cell–cell contacts, large increases in post-thaw recovery were observed, in contrast to suspension cryopreservation. This shows that chemically triggered extracellular ice nucleation using soluble nucleators mitigates intracellular damage by limiting the propagation of ice between adjacent cells and is a potent tool to improve the cryopreservation of complex cellular models.

To explore active ice nucleation for complex cellular model cryopreservation, we selected three common adherent cell lines; A549 (adenocarcinoma human alveolar basal epithelial), SW480 (human colon adenocarcinoma), and HepG2 (human Caucasian hepatocyte carcinoma). 2D monolayer cryopreservation is challenging with few technologies allowing significant cell recovery^44−46^ and hence is a stepping stone to cryopreservation of 3D spheroids (explored below). Suspension cryopreservation was also conducted, which allows segregation of the importance of cell–cell and cell–substrate contacts in monolayers (which can promote ice propagation) versus suspension (no cell–cell contacts). The active nucleating agent, from *Carpinus betulus* (Hornbeam) pollen, was prepared and sterilized as previously reported.^15^

Example ice nucleation droplet freezing data is included in the Supporting Information (Figure S1), demonstrating that the ice nucleators present in the pollen sample used are similar to those investigated previously, which were shown to raise the nucleation temperature in wells from −15 °C to −8 °C in the conditions used for cryopreservation in the present study.^15^

Figure 1 shows the 24 h post-thaw viability of cells after cryopreservation in 96-well plates. It should be reiterated at this point that cryopreservation in 96-well plates is extremely challenging due to supercooling of the aqueous solution and there are few examples of it being achieved.^14,16^ Cell recovery was measured both with 10% DMSO alone (−IN) and with induced ice nucleation (cryoprotectant containing 10% DMSO and PWW) (+IN). Previous work has suggested this nucleator increases the temperature of ice nucleation in wells which is significant in the cryobiological context.^15,18,47,48^ As would be expected, experiments show significant biological variability between individual measurements. To account for this variability and establish the statistical significance of our results we have analyzed our data using a mixed-effects model.^49^ The cells in suspension showed only small differences in post-thaw viability with stimulated ice nucleation (+IN), statistically significant only for HepG2 cells (Table S1), but the monolayers all showed significantly increased cell viabilities (*p* < 0.001 in all three cell lines; Table S1). This alone is an important observation that chemically inducing ice to form, in the extracellular media, can have such a major impact on the post-thaw viability of the cells. It also highlights that ice formation is not intrinsically detrimental in cryopreservation but that it does need to be controlled.

**Figure 1:**
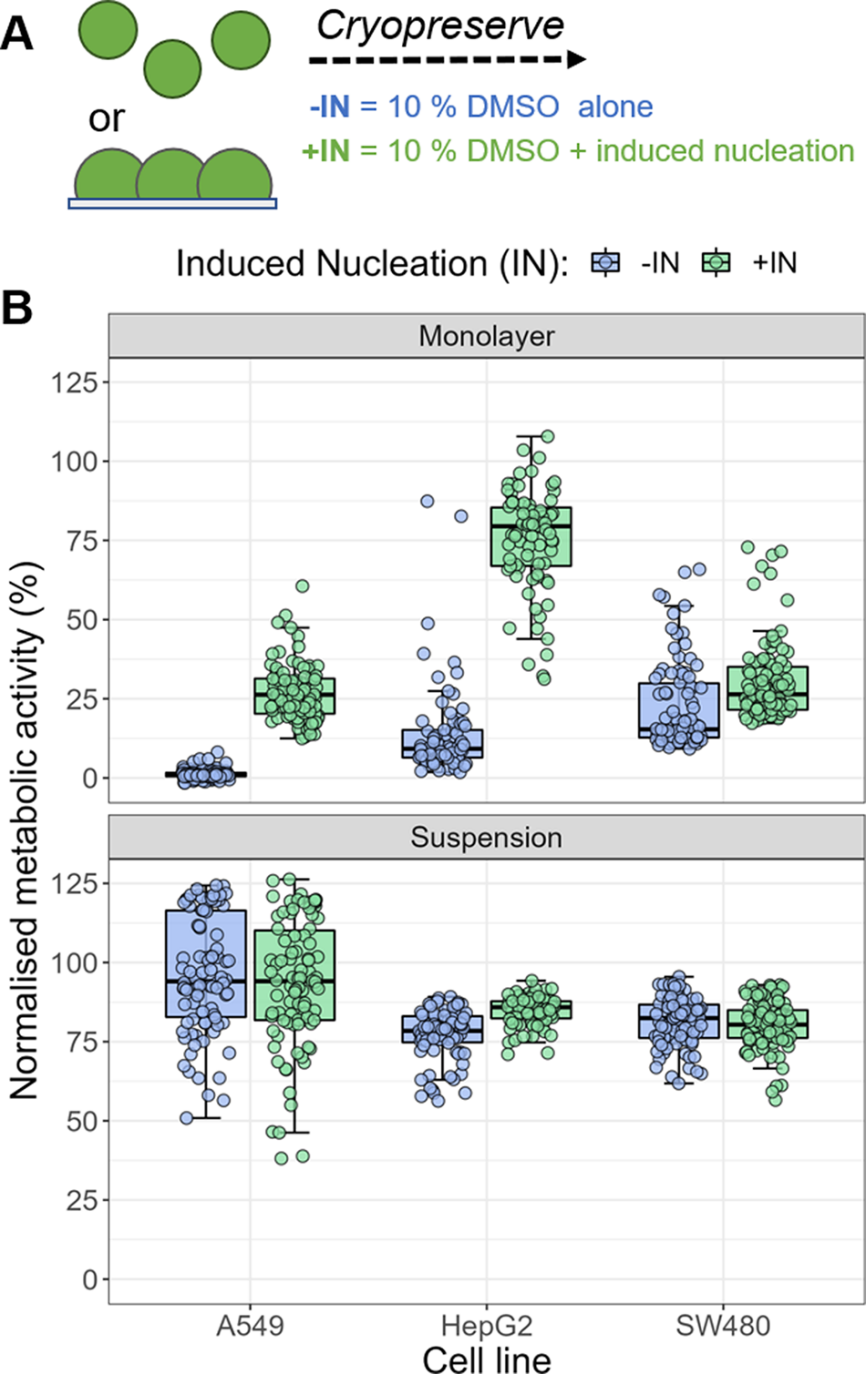
Cell viability 24 h post-thaw when frozen as monolayers or in suspension, with (+IN) or without (−IN) induced nucleation. (A) Schematic of cryopreservation format. (B) Viability of the three cell lines (A549, SW480, and HepG2) determined by metabolic activity assay (resazurin) 24 h post-thaw. Nonfrozen cells in the same format were used as controls.

The magnitude of improvement in post-thaw metabolic activity due to stimulated ice nucleation varied between the different adherent cell lines. Variation also existed between experimental replicates. Both observations are to be expected in biological systems. This variation was accounted for during statistical analysis via the application of a linear mixed effect models (see Supporting Information methods and results; Table S2).

Overall, these data raise the question: why does controlled ice nucleation aid cryopreservation of monolayers but not suspended cells? A key driver for cell death during cryopreservation is fatal intracellular ice formation (IIF). Damage to cells during cryopreservation can be caused by a range of mechanisms besides IIF,^50^ but it is clear that avoidance of IIF is critical. Acker et al. have shown that increasing nucleation temperature from −11 to −6 °C reduced the membrane damage to fibroblasts during cryopreservation, which correlated with fewer cells with intracellular ice (Figure 2A).^48^ It was also hypothesized that cells in monolayers are more likely to propagate ice between cells. Hence, controlling when, where and at what temperature the ice forms is crucial, compared to suspension cryopreservation where there are no cell–cell or cell–substrate contacts. To investigate this, we used cryomicroscopy to monitor ice formation in adherent cells during cooling. Ice formation could be seen on microscopy videos by darkening of the cells, due to increasing light scattering when frozen (Figure S2). Figure 2B shows the fraction of A549 cells suffering IIF, both with and without controlled ice nucleation. In the absence of controlled ice nucleation (−IN), typically 40–50% of the cells showed IIF, whereas controlled ice nucleation (+IN) reduced this to below 10% of cells. The magnitude of reduction is especially encouraging considering the nucleator is only applied external to the cells, yet controls an intracellular outcome, unlike solvent-based cryoprotectants that typically require permeation into the cell to be effective.

**Figure 2:**
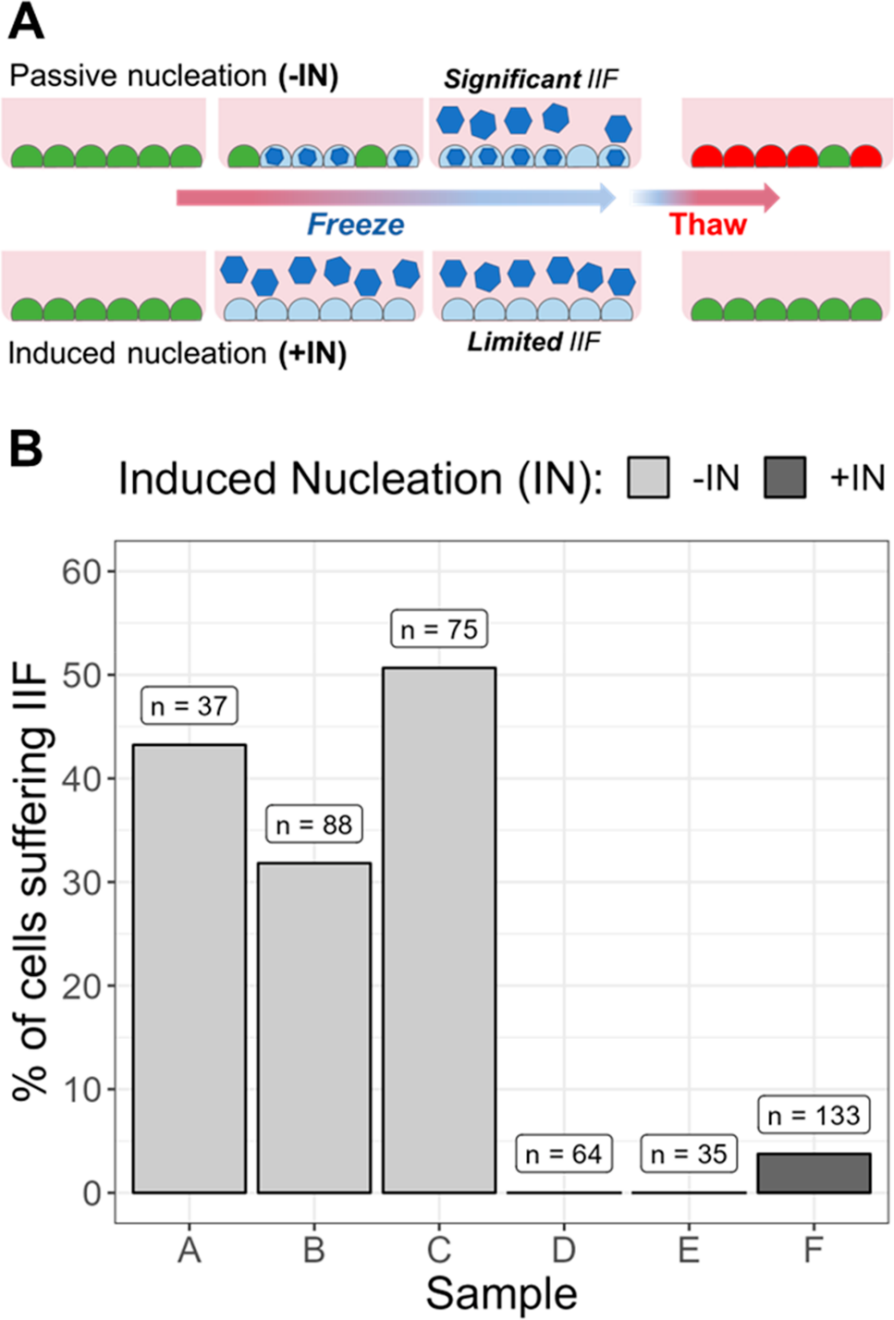
Degree of intracellular ice formation (IIF) in A549 cell monolayers with (+IN) and without (−IN) induced ice nucleation. (A) Schematic to show IIF formation in cell monolayers in the absence and presence of induced ice nucleation. (B) Fraction of cells experiencing IIF as determined by cryomicroscopy. Each condition (+IN or −IN) was repeated three times, and images were analyzed in ImageJ. Number of cells (*n*) measured is indicated above each bar.

With the above data showing that chemically induced ice nucleation improves the recovery of cell monolayers by reducing fatal IIF and reducing IIF propagation, we proceeded to spheroid (3D) cryopreservation. Spheroid cryopreservation is challenging because traditional cryoprotectants need to permeate to the core of the spheroids, without inducing toxicity, in addition to the IIF challenges we have described above and in Figure 2A. Spheroids also have extensive cell–cell contacts,^35^ which are known to transmit intracellular ice. Both A549 and HepG2 spheroids were prepared in U-bottom 96-well plates with either 4000 or 8000 cells/spheroid (Figure S3). Spheroids were cryopreserved in the plates to −80 °C in 10% DMSO, with or without stimulated ice nucleation. The spheroids were thawed, and allowed to recover for 24 h (to reduce false positives^51^), before evaluating viability by total ATP content, reported relative to the ATP content of cells before freezing. The ATP content assay is a convenient and effective method for assessing cell viability. [Note this method can report >100% viability as the cells are allowed to recover and grow post thaw].

Figure 3A shows the impact of induced extracellular ice nucleation on spheroid viability. As with previous 2D cryopreservation experiments, statistical analysis was conducted using linear mixed effect models (see Tables S3 and S4). This analysis showed that induced nucleation increased post-thaw viability from 26 to 55% (4000 cells) and 45 to 76% (8000 cells) for A549 spheroids. This result suggests that larger A549 spheroids may cope with the cryopreservation process better, even after normalization for prefreeze spheroid size. This finding is somewhat surprising as it would generally be anticipated that larger cell constructs would cope with cryopreservation less well. In comparison, in HepG2 cells no significant effect of spheroid size was found, resulting in an increase of cell viability of 50% (fixed effect estimates of 16 and 66% for −IN and +IN, respectively), independent of spheroid size (Table S5). For both cell lines, this is a substantial increase, achieved by changing ice nucleation temperature, rather than the cryopreservation formulation or freezing-rate profile. To provide further evidence of the efficacy of this chemically induced cryopreservation strategy, confocal microscopy of the thawed spheroids was undertaken. Using live (green)/dead (red) staining, it can be seen that for cryopreservation of A549 spheroids in 10% DMSO alone, there are many more red-labeled cells (membrane damaged) compared to when ice nucleation was induced (Figure 3B). Similar results were obtained for HepG2 cells (Figure S5). These observations support the hypothesis that IIF propagation between cells (which would damage membranes) is effectively mitigated by the induced ice nucleation.

**Figure 3:**
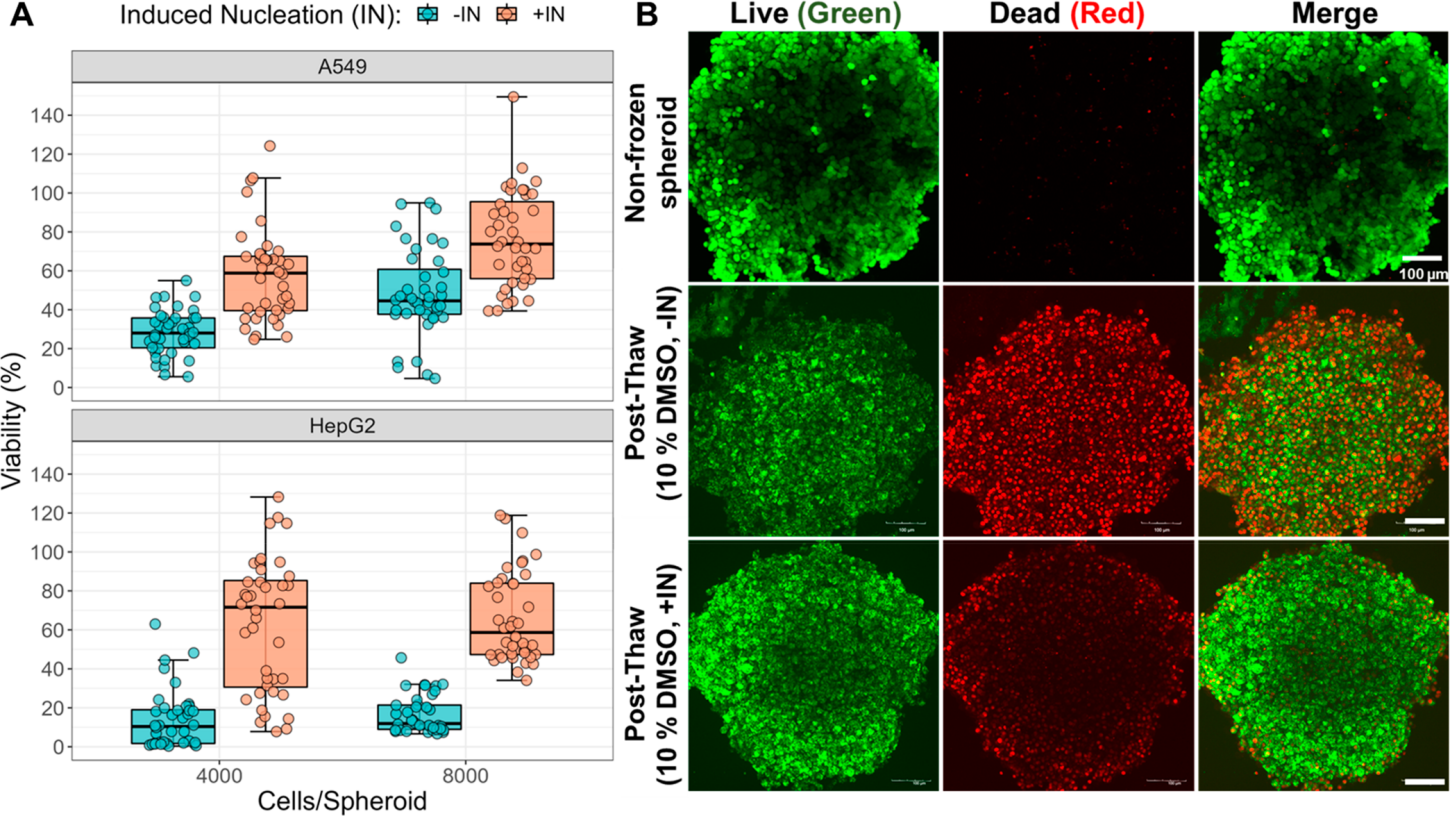
Spheroid viability 24 h post-thaw with (+IN) and without (−IN) active ice nucleation. (A) Post-thaw viability of A549 and HepG2 spheroids. Recovery determined by ATP content assay relative to nonfrozen spheroids of the same size. (B) Confocal microscopy of thawed A549 spheroids (8000 cells/spheroid) stained with live (green)/dead (red) assay. Brightness adjusted for display (consistent in a row) and all original images are in the Supporting Information (Figure S4).

An additional reactive oxygen species (ROS) assay was also conducted on the A549 spheroids (8000 cells/spheroid) (Figure 4)—ROS is a contributor to cell death post thaw, and it was observed here that with active ice nucleation ROS was decreased relative to DMSO alone. DMSO exposure^52^ and cryopreservation^53^ can both cause production of ROS. These data suggest that inducing ice nucleation decreases overall stress levels within the cells and that the mechanical process of ice nucleation can also mitigate biochemical damage, contributing to the high levels of recovery achieved here. It is worth noting that some spheroids frozen in the (−IN) condition were physically disrupted, as shown in Figure 4. This may be due to mechanical damage caused by rapid ice formation at low temperature in the absence on controlled ice nucleation.

**Figure 4:**
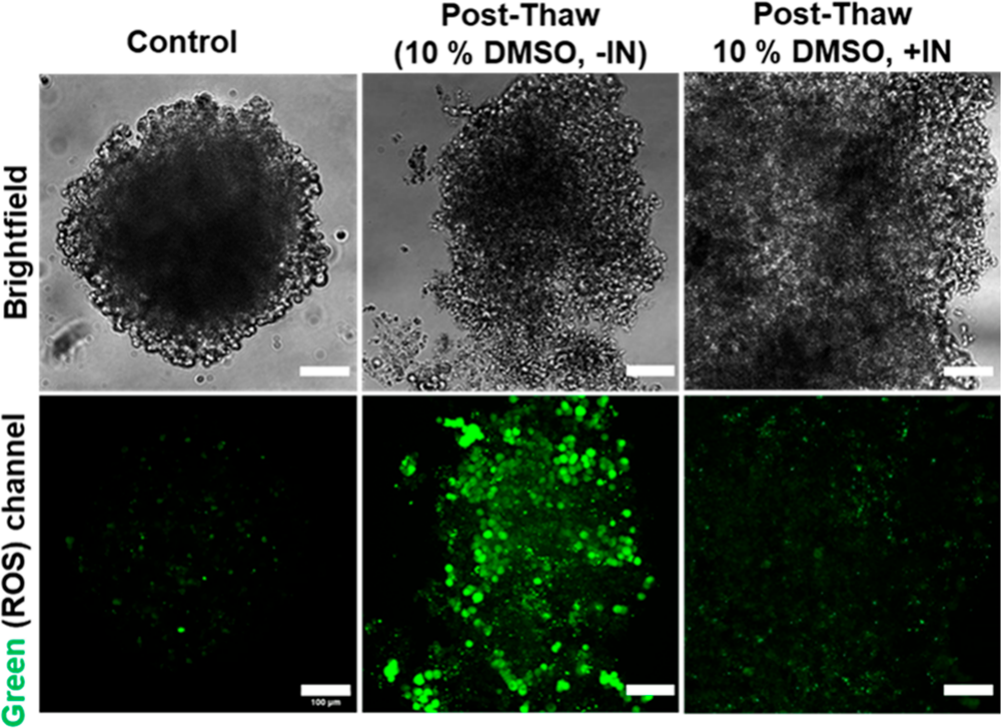
Reactive oxygen species analysis of A549 spheroids (8000 cells/spheroid). The control are nonfrozen spheroids. ROS was measured using dichlorodihydrofluorescein diacetate, producing green color to indicate ROS. Post-thaw measurements were after 24 h of culture. Scale bars are 100 μm.

Combined with monolayer and suspension cell data, these measurements support the hypothesis that soluble chemical nucleators can significantly protect multicellular structures during freezing by modulating the external environment to ensure ice forms at warmer (subzero) temperatures. This supports our early evidence that cell–cell and cell–substrate contacts lead to propagation of IIF and that by reducing the total fatal IIF by inducing warm-temperature ice nucleation, the cryopreservation of 3D constructs is dramatically improved.

In conclusion, we have demonstrated that soluble polysaccharide-based ice nucleators can reduce intracellular ice formation during DMSO-mediated cryopreservation. This reduction in intracellular ice allowed the routine cryopreservation and recovery of intact spheroids. Compared to DMSO alone, increases of 2–5-fold in cell recovery were observed. Triggered extracellular ice nucleation was shown to benefit scenarios with extensive cell–cell contacts (monolayers/spheroids) more than those without (suspension), supporting the hypothesis that intracellular ice can propagate between adjacent cells, and hence the need to prevent it. The active ice nucleation approach reduced the number of membrane damaged cells, and appeared to reduce overall reactive oxygen species, contributing to the exceptional post-thaw recovery results. The solubility and sterile nature of this chemical nucleator will enable its widespread use in 2D and 3D cryopreservation protocols, and the simplicity of addition to established cryopreservation solutions makes this easy to deploy compared to insoluble nucleators. While this study clearly demonstrates the efficacy of PWW as a nucleator for cryopreservation of cell-based models, it has recently been shown that warmer nucleation temperatures than those achieved using PWW will likely yield further improvements to cryopreservation outcomes, motivating further research into biocompatible ice nucleators.^18^ Altogether, this work shows that the design, discovery, and understanding of chemical ice nucleators is essential to enable banking and distribution of increasingly complex cell-based models. The easy storage of these models will increase their uptake in fundamental and applied studies, including to reduce the need for in vivo (animal) experimentation.

## Supporting Information Available

Complete experimental procedures including cell culture, pollen washing water preparation, details of statistical analysis, and ice nucleation data (PDF)
Movie showing ice formation (MP4)
