# Supplementary material for: Chemically Induced Extracellular Ice Nucleation Reduces Intracellular Ice Formation Enabling 2D and 3D Cellular Cryopreservation
Source: JACS Au. 2023 Apr 25;3(5):1314–20. doi: 10.1021/jacsau.3c00056 (PMC10207112; doi:10.1021/jacsau.3c00056)
Supplement: Supplementary file 1 — au3c00056_si_001.pdf [file au3c00056_si_001.pdf]

Supporting Information for

Chemically-Induced Extracellular Ice Nucleation Reduces Intracellular Ice Formation Enabling 2D and 3D Cellular Cryopreservation

Kathryn A. Murray,[a,b, ‡] Ya Nan Gao,[a,c, ‡], Christopher A. Griffiths,[d] Nina L. H. Kinney,[a] Qiongyu Guo,[c] Matthew I. Gibson[a,b*] and Thomas F. Whale[a*]

[a] Department of Chemistry, University of Warwick, Gibbet Hill Road, CV4 7AL, Coventry, UK,

[b] Division of Biomedical Sciences, Warwick Medical School, University of Warwick, Gibbet Hill Road, CV4 7AL, Coventry, UK,

[c] Department of Biomedical Engineering, Southern University of Science and Technology, Shenzhen, Guangdong 518055, China.

[d] Department of Aquatic Resources, Institute of Marine Research, Swedish University of Agricultural Sciences, Turistgatan 5, 453 30, Lysekil, Sweden

[‡]Authors contributed equally

Additional materials and methods

Preparation of pollen washing water (active ice nucleation agent).

Pollen washing water (PWW) was prepared as previously described.[1] Briefly, 0.2 g of pollen from hornbeam (*Carpinus betulus*) was suspended in 10 mL cell culture media overnight (see below for cell-line specific media) at 4 °C, then filtered through a 0.2 µm filter. It has previously been established that 32.8±1.1% of the mass of the pollen used is soluble.[7] This means that the final concentration of pollen derived material used is approximately 6.5 mg/ml. As discussed in Murray et al.[7] only a small fraction of this material is likely to consist of ice-nucleating macromolecules, with the balance made up by smaller saccharides.

Cell culture

Human Caucasian lung carcinoma (A549) cells (ECACC 86012804) were cultured in Ham's F-12K (Kaighn's) media (Gibco) supplemented with 10 % (v/v) foetal bovine serum (FBS, Sigma Aldrich), 100 units·mL^{-1} penicillin, 100 µg·mL^{-1} streptomycin, and 250 ng·mL^{-1} amphotericin B (1 % PSA, Gibco). Dukes' type B colorectal adenocarcinoma (SW480) cells (ECACC 87092801) were cultured in Advanced Dulbecco's Modified Eagle's Medium (DMEM, Gibco), supplemented with 10 % (v/v) FBS and 1 % PSA. Human liver hepatocellular carcinoma (HepG2) cells (ECACC 85011430) were cultured in Eagle's minimum essential media (EMEM, Merck) supplemented with 10% (v/v) FBS, 1% PSA and 1 % Gibco MEM non-essential amino acids 100X solution (1 % NEAA, Gibco). Cells were maintained in a humidified atmosphere at 37 °C, 5 % CO_2 and subcultured every 3-4 days. Cell dissociation was performed with 0.25 % trypsin with 1 mM EDTA in a balance salt solution. All well plates containing HepG2 cell monolayers were first coated with a Type I collagen solution (Merck).

Suspension and monolayer cryopreservation on 96 well plates

Cell cryopreservation in suspension and monolayer formats was performed as previously described and outlined in brief below.[1] For *monolayer cryopreservation*, cells were seeded into 96 well plates at 2.5×10^4 cells/well (A549, SW480) or 4.0×10^4 cells/well (HepG2) and allowed to adhere for 24 hours. A pre-freeze count was performed by dissociating and counting cells in control wells immediately prior to cryopreservation. 1× cryoprotectant solutions were prepared of either 10% DMSO in cell culture media (-IN) or 10% DMSO in 50% (v/v) PWW prepared with cell culture media (+IN). Cryoprotectant (50 µL) was added to each well and the plates were placed in a -80 °C freezer and allowed to cool at an uncontrolled rate. Plates were stored for 24 hours at -80 °C.

For *suspension cryopreservation*, cells were seeded into U-bottom 96 well plates at the same density as the immediate pre-freeze counts for monolayer cryopreservation (typically 4.0×10^4 cells in 25 µL for all cell types). Solutions of 2× cryoprotectant were prepared, consisting of either 20 % DMSO (-IN) or 20 % DMSO in 100 % (v/v) PWW (+IN), and 25 µL was added to each well (total well volume 50 µL). Final concentrations per well were 10 % DMSO or 10 % DMSO in 50 % PWW. Plates were placed in a -80 °C freezer and allowed to cool at an uncontrolled rate, then stored for 24 hours at -80 °C. The initial cooling rate generated by placing plates directly into the -80 °C freezer is 9 °C/min, as established in Murray et al.[1]

To thaw, all plates were removed from the freezer and 100 µL of warm (37 °C) media was added to each well. Plates were placed in an incubator at 37 °C for 10 minutes to ensure complete thawing. For monolayer cells, media was exchanged for fresh media then plates were incubated for 24 hours. Suspension plates were centrifuged at 730 *g* for 5 minutes to pellet cells. Media was replaced with fresh media, then the total well contents was transferred to a flat-bottom 96 well plate to facilitate adherent culture. Cells were cultured for 24 hours post-thaw before viability assessment by resazurin reduction assay.

A549 And HepG2 spheroid formation in low-attachment U-bottom 96-well plates

Cells were seeded at either 4000 or 8000 cells per well in the inner 60 wells of a low attachment U-bottom 96 well plate (Corning, CLS7007). Plates were centrifuged at 2000 rpm for 5 min then placed in an incubator (37 °C, 5% CO_2) for 5 days before cryopreservation to facilitate spheroid formation. Spheroids were monitored daily and spheroid diameter was measured from light microscopy images using imageJ, version 1.49.

Cryopreservation of spheroids in low-attachment 96 well plates

Spheroids were frozen in 50 µL of cryoprotectant (CPA) containing 10 % DMSO (-IN) or 10 % DMSO in 50 % (v/v) PWW (+IN), in U-bottom 96-well plates (Corning, NY). Plates were placed on a CoolCell® MP plate (BioCision, LLC, Larkspur, CA) and transferred to a -80 °C freezer, to allow a cooling rate of 1°C/min. After 24 h at -80 °C, the frozen plates were removed from the freezer and 100 µL of warm media was added to each well for rapid thawing. Plates were incubated at 37 °C for 3 min, then centrifuged and media exchanged for fresh media. Thawed plates were incubated for 24 hours post-thaw prior to analysis.

Evaluation of spheroid viability and morphology post-thaw

After spheroids cryopreservation and thawing, a CellTiter-Glo® 3D Cell Viability Assay (Promega, G9682, USA) was used to quantify ATP present in spheroids 24 h post-thaw. Non-frozen spheroids of the same size were initially analysed and served as a control group. Each frozen (24 h) and thawed (24 h) spheroid was transferred to individual wells of a white 96 well-plate, the assay was performed and the resulting luminescence was measured in a plate reader.

The LIVE/DEAD® Viability/Cytotoxicity Assay Kit was used to observe apoptotic cell death and morphology of spheroids. Samples (8000 cells/spheroid/well) were washed twice with a DPBS buffer and treated with 200 µL solution/well containing 2 µM of calcein AM (5 µL) and 4 µM of ethidium homodimer-1 (20 µL) in sterile DPBS for 1 hour and then transferred to confocal dishes (VWR International Ltd., UK, 734-2904). Spheroids were imaged using a FV3000 confocal laser-scanning microscope (Olympus, Tokyo, Japan). The polyanionic dye calcein AM retained within live cells, showing an intense uniform green florescence in live cells at ex/em ~495 nm/ ~515 nm. EthD-1 entered cells with damaged membranes, binding to nucleic acids and producing a bright red fluorescence in dead cells at ex/em ~495 nm/ ~635 nm.

Reactive oxygen species (ROS) assay

Reactive Oxygen Species detection reagent (Invitrogen, D399) was both before and after-freezing spheroids (8000 cells/spheroid/well) imaging. Samples were washed twice with a DPBS buffer and incubated with 200 µL/well dichlorodihydrofluorescein diacetate (DCFDA) solution (20 µL of 7.5 mM diluted stock in PBS) for 30 minutes and then transferred to confocal dishes. The FV3000 confocal laser-scanning microscope (Olympus, Tokyo, Japan) was applied to image the spheroids with fluorescence excitation and emission in 492-495/505 and 517-527 nm.

Statistical analysis

Linear mixed effect (LME) models were used to test for the effect of ice nucleation (induced (+IN) vs passive (-IN)) on all three cell lines (A459, HepG2 and SW480) and cryopreservation formats (monolayer, suspension and spheroids). The response variables considered were normalised metabolic activity (nMA) in the monolayer and suspension formats, and normalised viability in the spheroid format. In both cases, nMA and viability are reported as percentages. In each case, several explanatory variables were considered and were coerced to factors prior to model fit. All LME models were fit using restricted maximum likelihood (REML) and were used to ensure that the variability associated with experimental replicates could be appropriately considered in the random effects distribution of the

model. In each case, stepwise model selection was conducted using ANOVA and Akaike information criterion (AIC). Model fit was assessed via visual inspection of the residuals. Models were fit separately to each cell line (A549, HepG2 and SW480) and to monolayer/suspension and spheroid, respectively. All models were fitted in R (R Core Team, 2021; version 4.1.2)[2] using the lme4 package.[3]

Data manipulation and visualisation made full use of the tidyverse collection of R packages[4] in particular the dplyr[5] and ggplot2[6] packages. Color palettes were sourced from the rcartocolor package.[7]

Additional data

Droplet Ice Nucleation Measurements

Ice nucleation temperatures of microlitre droplets were measured using a custom-built droplet freezing assay, as described previously.[1] The hornbeam pollen solution for nucleation measurements was prepared by adding 0.04 g *Carpinus betulus* pollen, purchased from Pharmallerga®, to 2 ml Milli-Q® water. The pollen suspension was refrigerated overnight before filtering through a 0.2 µm syringe filter into a clean glass vial. For the ice nucleation measurements, 40-50 microlitre droplets of the filtered, sterile pollen solution were pipetted, using a Sartorius Picus® electronic micropipette, on to a 22 mm diameter Hampton Research HR3-231 siliconized glass slide, placed on the aluminium plate of the cold stage. The cold stage temperature was lowered at a rate of 2 °C/min and the droplet freezing temperatures recorded. Nucleation temperatures of 1 µL Milli-Q® water droplets were measured using the same set-up for comparison. This demonstrates that the Hornbeam pollen used contains ice nucleators of the type used in our previous study on the use of pollen ice nucleators for cryopreservation.

Figure S1 shows the ice nucleation data produced for this study and ice nucleation data for 1 µl droplets of *Carpinus betulus* PWW reported in Murray et al.[7] It can be seen that the *Carpinus betulus* PWW sample produced here has very similar ice nucleation properties to the sample used previously. As such, we conclude that the larger volumes used for cryopreservation in 96-well plates will freeze at similar temperatures to those found in Murray et al.[7] using thermocouples embedded in 96-well plates. For comparison, Figure S1 also shows the data produced there for freezing of 100 µl volumes of PWW in 96-well plates.

Direct and accurate measurement of the ice nucleation temperatures in 96-well plates is challenging, and has only recently been accomplished using unique, bespoke infrared thermometry instruments.[11,12] By using 3 µl droplets we show that a larger fraction of droplets tends to freeze at warmer temperatures as increased volume of PWW is used, due to the presence of a rarer, more active ice nucleating macromolecules. In Murray et al.[7] it was estimated that Hornbeam PWW raises the nucleation temperature in 50 µl volumes of DMSO cryopreservation media to -8°C, high enough to expect substantially improved cryopreservation outcomes according to literature data.[4,5] We expect to see the same shift in nucleation temperature in this study. Future work should investigate the freezing temperatures of 50 µl volumes of PWW using infrared thermography.[11,12]

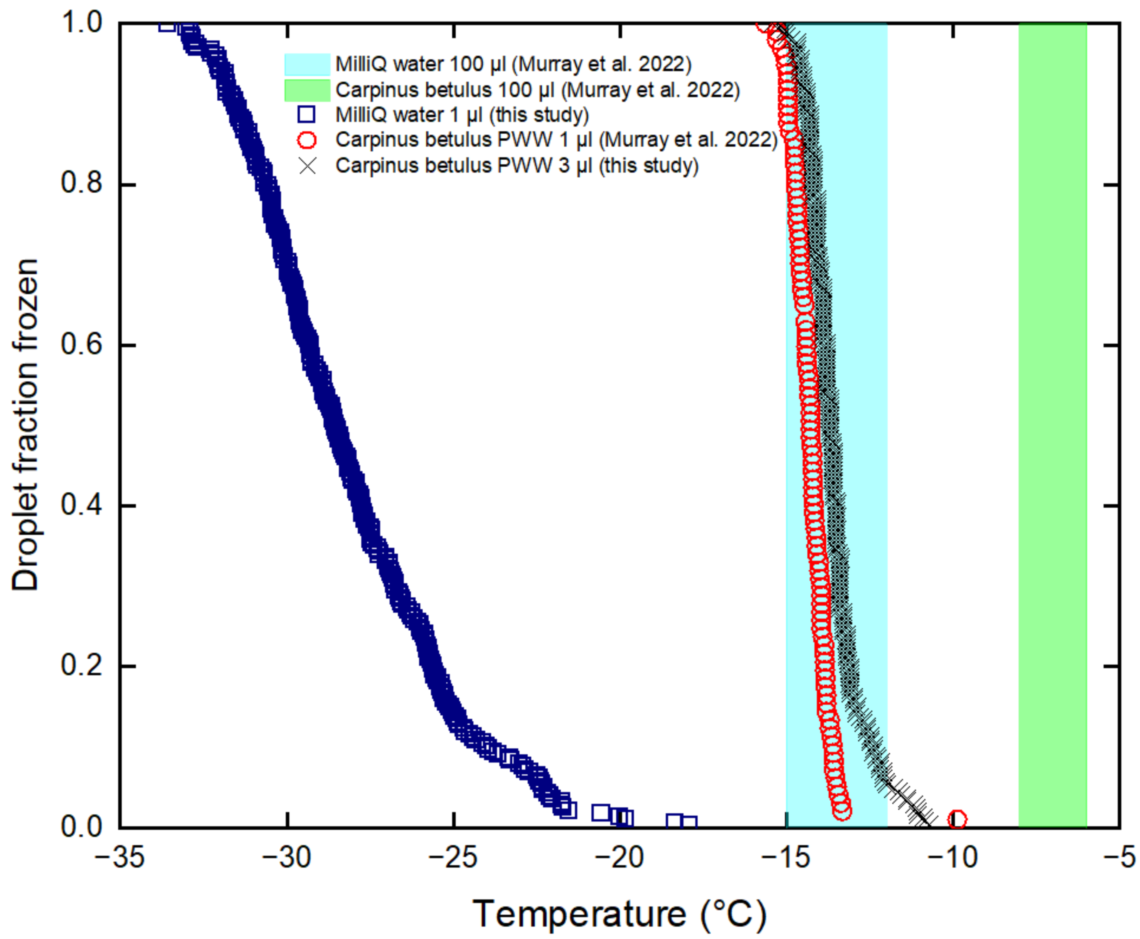

Figure S1. Droplet fraction frozen for microlitre scale droplets of *Carpinus betulus* PWW (246 droplets) and Milli-Q® water (273 droplets). Also included is literature data from Murray et al.[7] for similar droplets, along with estimated freezing temperature ranges for pure water and *Carpinus betulus* PWW (shaded regions) produced using thermocouples embedded in 96-well plates to detect the latent heat of freezing.

Cryomicroscopy of A549 monolayer cryopreservation

The data in Figure 2B of the main text were produced by cryomicroscopy of the freezing of A549 cell monolayers grown on glass coverslips. The approach taken was inspired by work by Acker et al.[8] Cells were grown in the manner described in the 'cell culture' section above before being seeded onto circular glass coverslips placed on the bottom of 12-well plates, and allowed to adhere for 24 hours. To perform experiments, the cell culture media was removed and 20 µl of either 10% DMSO in cell culture media (-IN) (3 experiments) or 10% DMSO in 50% (v/v) PWW prepared with cell culture media (+IN) (3 experiments) added on top of the cell monolayer. The cryoprotectant solutions spread out to form a thin liquid layer. The coverslips were then quickly transferred to a Linkam Cryostage BCS196 and cooled rapidly from room temperature to 0°C, then at a rate of 2°C/min to -40°C while a Canon DSLR 500D digital camera equipped to an Olympus CX 41 microscope with a UIS-2 20x/0.45/∞/0-2/FN22 lens was used to take video of the cells. While the experimental setup did not allow accurate determination of ice nucleation temperatures, freezing began below -10°C in all -IN experiments and above -10°C in all +IN experiments.

After ice nucleation occurred, ice was seen to rapidly grow across the entire visible image. Darkening of individual cells, taken to indicate intracellular ice formation (IIF), could be observed through the ice layer. Individual cells darkened over the course of about 10 frames (around half a second). Cells darkened at different times, with events taking place over a span of 10-15 seconds after initial ice formation.

ImageJ v1.49 was used to create an overlay of cell locations, and the number of cells in each experimental frame counted. The overlay was used to correlate darkening events with cell locations, allowing the fraction of cells experiencing IIF reported in Figure 2B to be determined. Only cells which were adhered to the surface were included. Figure S2 and Movie S1 show the cryomicroscopy process. Movie S1 shows both the IN- and IN+ conditions. While the observed changes in contrast are subtle, they are clear on video, and allow reasonably straightforward assessment of the proportion of cells darkening during cryopreservation. In two of the three experiments performed with control of ice nucleation no cells at all were observed to darken, while darkening was observed in many cells in the experiments where nucleation was not controlled. In all cases, there was a tendency for darkening of a cell to immediately precede darkening of neighbouring cells, in line with previous observations.[8] At the conclusion of each experiment the cells were rewarmed. In all cases the appearance of the cells was unchanged from the original pre-freezing state, although occasional non-adhered cells tended to move.

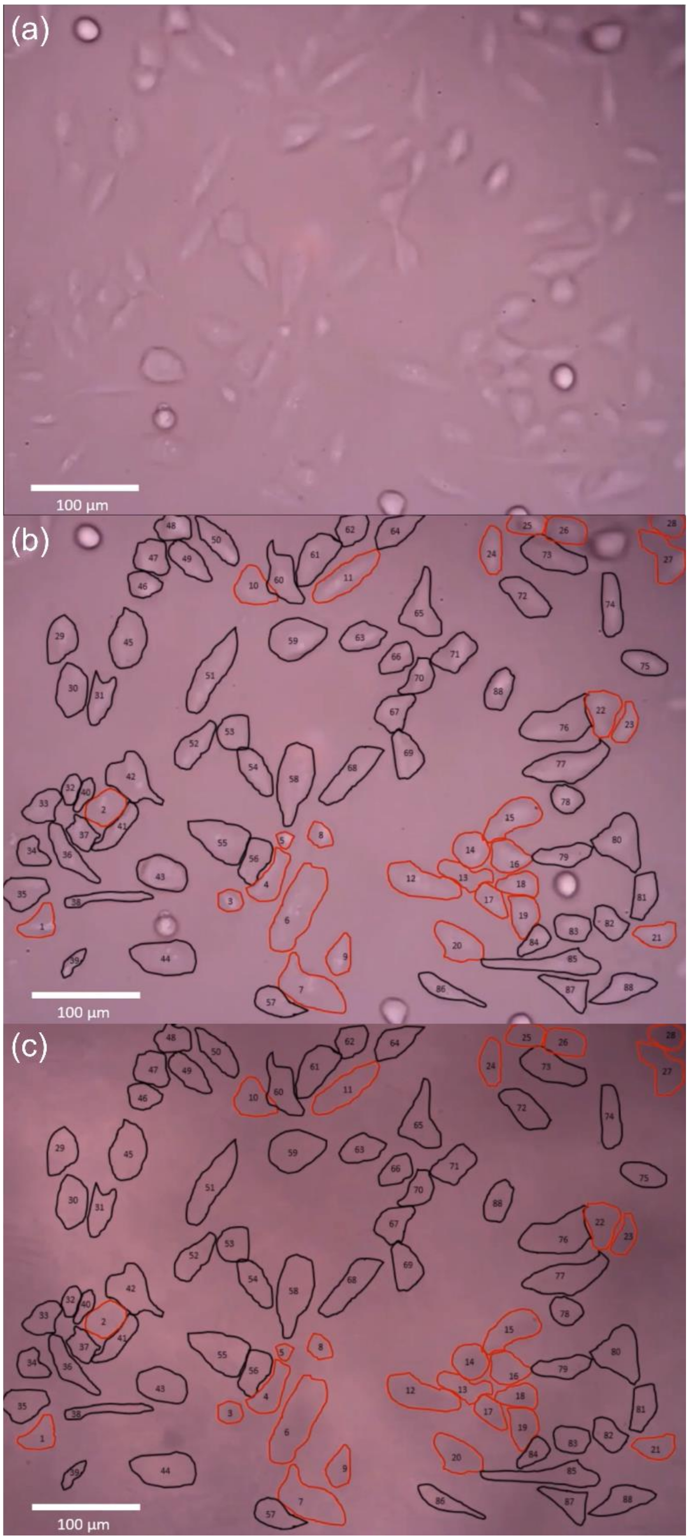

Figure S2: Micrographs of A549 cell monolayer undergoing cryopreservation in the absence of controlled nucleation (-IN). (A) Monolayer pre-freezing. (B) Monolayer pre-freezing with cell positions labelled. (C) Monolayer after formation of the ice layer. Darkening of individual cells can be observed through the thin layer of ice. Red cells (numbers 1-28) darken (indicating IIF) in the course of the experiment. Black cells (numbers 29-88) do not.

Statistical analysis by cell line – monolayer/suspension

A549. Induced ice nucleation was found to have a significantly positive effect on the normalised metabolic activity (nMA) of A549 cells post-thaw in the monolayer format (p-value < 0.001; Figure 2). In comparison, no effect of induced ice nucleation on nMA was found in the suspension format (p-value = 0.29; Figure 2). Model selection via AIC demonstrates that the most parsimonious model includes a fixed effect interaction term between ice nucleation and cell format, as well as a random intercept term for experimental replicate (Table S2). This linear mixed effect model is found to explain 93% (R squared = 0.93; Table S2) of the variance in nMA. The model shows that both format and the variability associated with experimental replicate do have an effect on the relationship between ice nucleation and nMA. Model estimates show that the nMA is, on average, 26% higher in the monolayer format when ice nucleation is induced compared to passive (Table S1). They also show that nMA is higher in the suspension format independent of test conditions (Table S1).

HepG2. Induced ice nucleation was found to have a significantly positive effect on the nMA of HepG2 cells post-thaw in both formats (p-value < 0.001 for both monolayer and suspension; Figure 2). Model selection via AIC demonstrates that the most parsimonious model includes a fixed effect interaction term between ice nucleation and cell format, as well as a random intercept term for experimental replicate (Table S2). This linear mixed effect model is found to explain 86% (R squared = 0.86; Table S2) of the variance in nMA. The model shows that both format and the variability associated with experimental replicate do have an effect on the relationship between ice nucleation and nMA. When ice nucleation is induced as opposed to passive, model estimates show that nMA is, on average, 61% and 8% higher in the monolayer and suspension formats, respectively (Table S1). They also show that nMA is higher in the suspension format (Table S1).

SW480. Induced ice nucleation was found to have a significantly positive effect on the nMA of SW480 cells post-thaw in the monolayer format (p-value < 0.001; Figure 2). In comparison, no effect of induced ice nucleation is found in the suspension format (p-value = 0.38; Figure 2). Model selection via AIC demonstrates that the most parsimonious model includes a fixed effect interaction term between ice nucleation and cell format, as well as a random intercept term for experimental replicate (Table S2). This linear mixed effect model is found to explain 88% (R squared = 0.88; Table S1) of the variance in nMA. The model shows that both format and the variability associated with experimental replicate do have an effect on the relationship between ice nucleation and nMA. Model estimates show that the nMA is, on average, 8% higher in the monolayer format when ice nucleation is induced compared to passive (Table S1). They also show that nMA is higher in the suspension format independent of test conditions (Table S1).

Table S1. Fixed effect estimates and confidence intervals (lower = 2.5% - higher = 97.5%) for normalised metabolic activity (%) extracted from the most parsimonious models for each cell line (see Table 2). All values are rounded to two decimal places.

		**Format**	
**Cell line**	**Ice Nucleation**	*Monolayer*	*Suspension*
A549	+IN	26.94<br>(9.91 – 43.98)	93.30<br>(76.27 – 110.34)
	-IN	1.20<br>(-12.41 – 14.81)	96.36<br>(82.74 – 109.97)
HepG2	+IN	74.91<br>(66.83 – 82.91)	84.48<br>(76.40 – 92.48)
	-IN	13.43<br>(8.98 – 17.80)	76.68<br>(72.23 – 81.05)
SW480	+IN	30.56<br>(21.77 – 39.35)	80.20<br>(71.41 – 88.99)
	-IN	22.42<br>(16.63 – 28.21)	81.21<br>(75.42 – 87.00)

Table S2. Model selection table via AIC for all three cell lines (A459, HepG2 and SW480) for monolayer and suspension cryopreservation. ΔAIC values are reported as differences from the most simplistic model. The most parsimonious model is shown in bold. R2 squares are reported as the conditional (including both fixed and random effects) R squared and are calculated using the r.squared.GLMM function in the 'MuMIn' package in R.[9]

**Cell line**	**Model formula**	**AIC**	**ΔAIC**	**R^2 value**
A549	nMA ~ (1|Replicate)	3743.4	-	0.05
	nMA ~ IN + (1|Replicate)	3739.1	-4.3	0.06
	nMA ~ Format + (1|Replicate)	2984.2	-759.2	0.89
	nMA ~ IN + Format + (1|Replicate)	2929.6	-813.8	0.90
	**nMA ~ IN * Format + (1|Replicate)**	**2815.8**	**-927.6**	**0.93**
HepG2	nMA ~ (1|Replicate)	3030.4	-	0.00
	nMA~ IN + (1|Replicate)	2913.9	-116.5	0.32
	nMA ~ Format + (1|Replicate)	2898.4	-132.0	0.35
	nMA ~ IN + Format + (1|Replicate)	2692.3	-338.1	0.67
	**nMA ~ IN * Format + (1|Replicate)**	**2428.3**	**-602.1**	**0.86**
SW480	nMA ~ (1|Replicate)	3459.2	-	0.13
	nMA ~ IN + (1|Replicate)	3459.9	0.7	0.17
	nMA ~ Format + (1|Replicate)	2739.2	-720.0	0.87
	nMA ~ IN + Format + (1|Replicate)	2730.9	-728.3	0.87
	**nMA ~ IN * Format + (1|Replicate)**	**2715.3**	**-743.9**	**0.88**

Statistical analysis by cell line – spheroids

A549. Induced ice nucleation was found to have a significantly positive effect on the viability (V) of A549 cells post-thaw in the spheroid format (p value = < 0.001; Figure 3). This positive effect was found to occur in both sizes (4000 vs. 8000). We also find that a larger number of cells (8000) significantly increased viability. Model selection via AIC demonstrates that the most parsimonious model includes fixed effect terms for ice nucleation and size, however, an interactive term between the two explanatory variables is not supported (Table S3). This lack of support suggests that the expected positive effect of induced ice nucleation on viability is expected to be the same in either size. This linear mixed effect model is found to explain 56% (R squared = 0.56; Table 3) of the variance in viability. Model estimates show that the viability is, on average, 29% higher under induced ice nucleation compared to passive ice nucleation (Table S4).

HepG2. Induced ice nucleation is found to have a significantly positive effect on the viability of cells post-thaw in the spheroid format (p value = < 0.001; Figure 3). This positive effect is found to occur in both sizes (4000 vs. 8000) but we find no significant effect of size on viability meaning that the positive effect of induced nucleation is independent of size. Model selection via AIC demonstrates that the most parsimonious model includes a single fixed effect term for ice nucleation (Table S3). This linear mixed effect model is found to explain 71% (R squared = 0.71; Table 3) of the variance in viability. Model estimates show that the viability is, on average, 49% higher under induced ice nucleation compared to passive ice nucleation (Table S5).

Table S3. Model selection table via AIC for spheroid experiments. ΔAIC values are reported as differences from the most simplistic model. The most parsimonious model is shown in bold. R2 squares are reported as the conditional (including both fixed and random effects) R squared and are calculated using the r.squared.GLMM function in the 'MuMIn' package in R.[9]

Cell line	Model formula	AIC	ΔAIC	R^2 value
A549	V ~ (1|Replicate)	1506.0	-	0.18
	V ~ IN + (1|Replicate)	1447.3	-58.7	0.45
	V ~ Size + (1|Replicate)	1483.9	-22.1	0.30
	**V ~ IN + Size + (1|Replicate)**	**1412.3**	**-93.7**	**0.56**
	V ~ IN * Size + (1|Replicate)	1414.3	-91.7	0.56
HepG2	V ~ (1|Replicate)	1563.6	-	0.18
	**V ~ IN + (1|Replicate)**	**1402.6**	**-161.0**	**0.71**
	V ~ Size + (1|Replicate)	1565.1	+1.5	0.18
	V ~ IN + Size + (1|Replicate)	1403.3	-160.3	0.71
	V ~ IN * Size + (1|Replicate)	1405.3	-158.3	0.71

Table S4. Fixed effect estimates and confidence intervals (lower = 2.5% **-** higher = 97.5%) for viability (%) of A549 cells extracted from the most parsimonious models (see Table 3). All values are rounded to two decimal places.

	**Size**	
**Ice Nucleation**	*4000*	*8000*
+IN	55.00 (31.87 – 77.96)	74.27 (51.13 – 97.23)
-IN	26.09 (8.84 – 43.17)	45.36 (28.11 – 62.43)

Table S5. Fixed effect estimates and confidence intervals (lower = 2.5% **-** higher = 97.5%) for viability (%) of HepG2 cells extracted from the most parsimonious models (see Table 3). All values are rounded to two decimal places.

**Ice Nucleation**	**Estimate**
+IN	65.67 (40.35 – 90.84)
-IN	16.45 (-3.04 – 36.00)

Formation of A549 spheroids

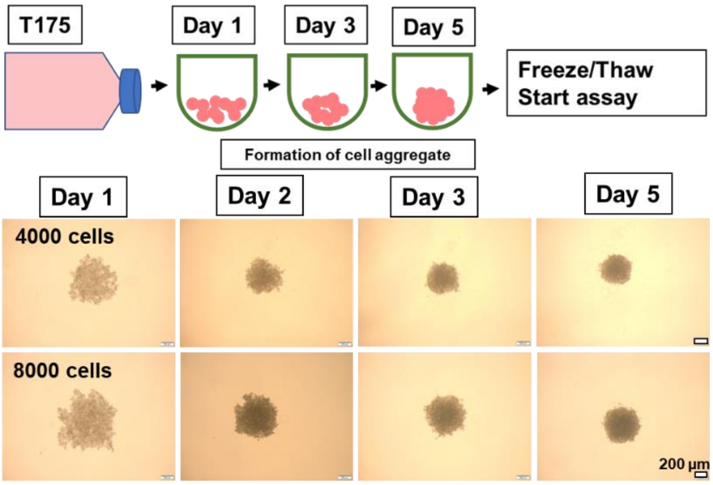

Figure S3. Spheroids preparation. A549 cells were seeded in the inner 60 wells of a low-attachment 96-well plate, and grown for five days.

Original microscopy images of spheroids in figure 3

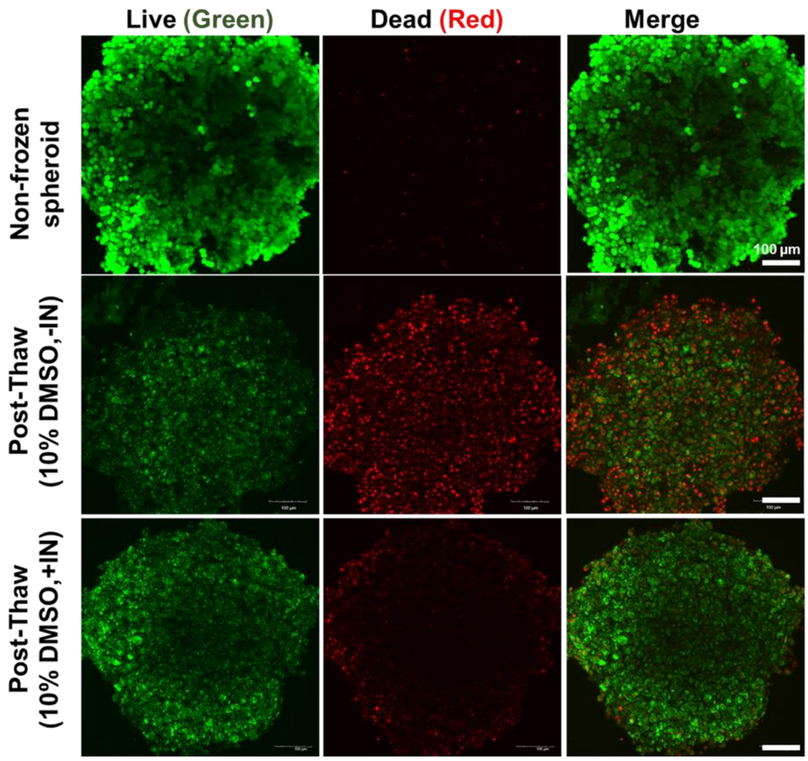

Figure S4. Confocal microscopy of thawed A549 spheroids (8000 cells/spheroid) stained with Live (green)/Dead (red) assay, scale bar: 100 µm. Original images. In the main paper the images in every row were color adjusted identically.

Confocal microscopy of HepG2 spheroids post-thaw

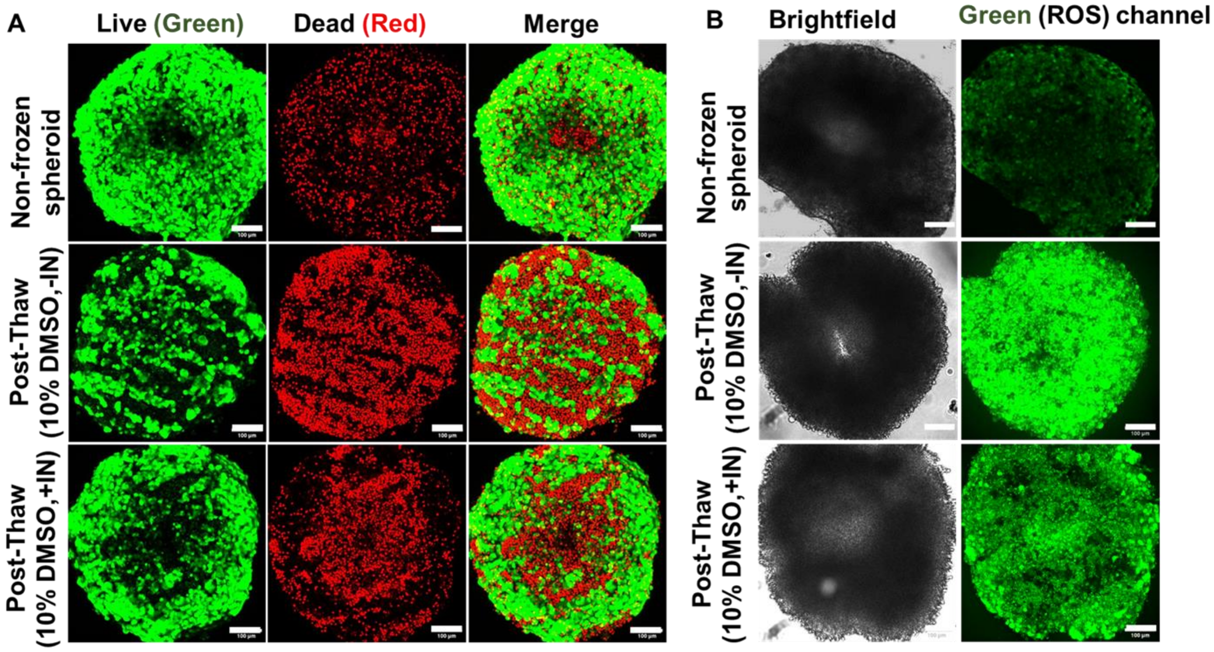

Figure S5. Confocal microscopy of before and post-thawed HepG2 spheroids (8000 cells/spheroid) stained with Live (green)/Dead (red) assay (A). Reactive oxygen species (ROS) analysis of HepG2 spheroids using dichlorodihydrofluorescein diacetate producing green color (B). The control are non-frozen spheroids. scale bar: 100 µm.

References

(1) Murray, K. A.; Kinney, N. L. H.; Griffiths, C. A.; Hasan, M.; Gibson, M. I.; Whale, T. F. Pollen Derived Macromolecules Serve as a New Class of Ice-Nucleating Cryoprotectants. *Sci. Rep.* **2022**, *12* (1), 12295.

(2) R Core Team: A language and environment for statistical computing. R Foundation for Statistical Computing, Vienna, Austria. **2021**, https://www.r-project.org/

(3) Bates, D.; Mächler, M.; Bolker, B. M.; Walker, S. C. Fitting Linear Mixed-Effects Models Using Lme4. *J. Stat. Softw.* **2015**, *67* (1), 1–48.

(4) Wickham, H.; Averick, M.; Bryan, J.; Chang, W.; McGowan, L.; François, R.; Grolemund, G.; Hayes, A.; Henry, L.; Hester, J.; Kuhn, M.; Pedersen, T.; Miller, E.; Bache, S.; Müller, K.; Ooms, J.; Robinson, D.; Seidel, D.; Spinu, V.; Takahashi, K.; Vaughan, D.; Wilke, C.; Woo, K.; Yutani, H. Welcome to the Tidyverse. *J. Open Source Softw.* **2019**, *4* (43), 1686.

(5) Wickham, H.; Francois, R.; Henry, L.; Muller, K. dplyr: A Grammar of Data Manipulation. (R package version 1.0.9). **2022**, https://cran.r-project.org/package=dplyr

(6) Wickham, H. ggplot2: Elegant Graphics for Data Analysis. Springer-Verlag New York. **2016**, https://ggplot2.tidyverse.org

(7) Nowosad, J. "CARTOColors" Palettes. **2018**, https://nowosad.github.io/rcartocolor

(8) Acker, J. P.; Larese, A.; Yang, H.; Petrenko, A.; McGann, L. E. Intracellular Ice Formation Is Affected by Cell Interactions. *Cryobiology* **1999**, *38* (4), 363–371.

(9) Bartoń, K.; MuMIn: Multi-Model Inference. R package version 1.43.17. **2020**. https://CRAN.R-project.org/package=MuMIn
